# Supplementary material for: The Future of Careers at the Intersection of Climate Change and Public Health: What Can Job Postings and an Employer Survey Tell Us?
Source: Int J Environ Res Public Health. 2020 Feb 18;17(4):1310. doi: 10.3390/ijerph17041310 (PMC7068354; doi:10.3390/ijerph17041310)
Supplement: Supplementary file 1 [file ijerph-17-01310-s001.pdf]

International Journal of Environmental Research and Public Health

MDPI

Article

The Future of Careers at the Intersection of Climate Change and Public Health: What Can Job Postings and an Employer Survey Tell Us?

Heather Krasna [1,2,*], Katarzyna Czabanowska [2,3,4], Shan Jiang [1], Simran Khadka [1], Haruka Morita [1], Julie Kornfeld [1] and Jeffrey Shaman [1]

Supplementary Materials S1: Survey Questions

Climate and Health Jobs of the Future Survey

Start of Block: Default Question Block

Q1 What is your name?

____

Q2 What is your organization's name?

____

Q3 What is your job title?

____

Q4 What is your email address?

____

Q5 Has your organization hired people with a Master of Public Health or PhD in Public Health in the past?

○ Yes (1)

○ No (2)

○ Don't know (3)

Q6 Would any of the following skills be useful to your organization?

	**Extremely Useful (1)**	**Moderately Useful (2)**	**Slightly Useful (3)**	**Neither Useful Nor Useless (4)**	**Slightly Useless (5)**	**Moderately Useless (6)**	**Extremely Useless (7)**
Health Impact Assessment (1)	○	○	○	○	○	○	○
GIS Mapping (2)	○	○	○	○	○	○	○
SAS (3)	○	○	○	○	○	○	○
R (4)	○	○	○	○	○	○	○

Risk Assessment (5)							
Knowledge of climate, carbon and water cycles (6)							
Familiarity with climate modeling approaches (7)							
Knowledge of climate mitigation, adaptation, and climate-health co-benefits (8)							
Familiarity with climate-health justice issues (9)							
Knowledge of direct, indirect and downstream effects of climate on health (10)							
Understanding pollution - health consequences, causes and sources (11)							
Exposure science methods (12)							
Epidemiological methods (13)							
Dynamic model approaches to climate and health (14)							
Other (please list) (15)							

Q7 Do you expect the need to hire people with a background in climate and public health to grow in your organization in the next 5–10 years?

○ Yes (1)

○ Maybe (2)

○ No (3)

○ Don't know (4)

○ Q8 What expertise or skills do you think will be needed to address the issue of climate change and human health in the next 10–20 years?

____

End of Block: Default Question Block

Supplementary Materials S2: Example Quotes from Employer Survey

1. 1. "Broad, widely applicable skills such as finance and budgeting, communication, writing, the ability to influence without authority, etc. are the types of skills that can augment knowledge of climate change and health, especially in programmatic positions at nonprofits."

2. "Big Data Management and Analysis, Environmental Economics/valuation of non-market goods, Ecological design/principles of ecosystem resilience."
3. "Ability to work across sectors (health, education, housing, private, public, etc.), experience or knowledge of a variety of financing schemes"
4. "Understanding and addressing the underlying conditions that make people more at-risk to climate related health impacts"
5. "Systems thinking, racial justice and inequity of all types, social sciences and human behavior"
6. "Behavioral change, mental health and climate change, transformational resilience"
7. "Increasingly a "triple bottom line" concept is growing in importance, and indicators such as health benefits, reduced asthma rates and other indicators are being quantified and considered when investing in energy efficiency or renewable energy projects."
8. "Being able to bake climate change mitigation strategies into existing government processes of long-term planning, land acquisition, landscape design, and construction. Human-centered design."
9. "Knowledge of agricultural impact of climate change; ideas on addressing nutrient deficiency amidst chronic natural disasters and crop failure, new agricultural techniques for disaster risk reduction"
10. "Climate change communications, movement building, disaster preparedness, mental health support related to climate issues"
11. "Organizational, Planning, GIS, TIC's, Big Data Platforms access and knowledge, Lab analysis and interpretation, Creation, Code and Registration of Data."
12. "data management and analytical skills; knowledge of the general principles of health education and/or environmental health; knowledge of SAS and/or ArcGIS software; Understanding connections between local public health, community planning, natural resources, and environmental justice; engaging stakeholders from diverse communities; facilitating multi-disciplinary work groups"
13. "an ability to leap beyond sustainability concepts into true resilience - for instance, ensuring climate-migrant receiving communities are places of restorative justice where the social safety net is ready to handle those traumatized by the move. This is universal need - Louisiana to Bangladesh."
14. "Methodology for quantifying the public health and health equity benefits (e.g., premature deaths avoided, disease burden reduced, health care cost savings, etc.) associated with climate mitigation and adaptation strategies and programs. For example, how effective are our state climate programs and investments for clean mobility/transportation, affordable housing, sustainable communities, workforce development, etc. in producing health and equity benefits?"
15. "Climate adaptation strategies; understanding of funding opportunities for infrastructure investment; GIS mapping of energy use/GHG/flooding; hydrology and hydrogeology"
16. "We need a paradigm sheet in developing a holistic curriculum at all levels which fully integrates in great depth the nexus between climate change and human health."
17. "Courses that include economic assessment of alternatives proposed for climate change adaptation and mitigation."
18. "Data Analysis, Satellite imagery, epidemiology"
19. "Legislative writing skills"
20. "Experience observing/tracking climate change related migration"
21. "Cross disciplinary understanding of food systems, economic development, human health (including genomics, microbiome), botanics (biodiversity), soil health"
22. "Incorporating the relevant evidence into actual policy, something that's sorely lacking today."
23. "Mental health and psychosocial impacts will be far more widespread and last far longer than physical health impacts-- prevention and treatment methods-especially prevention-will be the most critical needs, though few people in the public health field have grasped this yet."
24. "Need effective fundraisers. Also need to train/develop workforce and get local funding for climate-health ambassadors."
25. "Cost benefit analysis; valuation of "non market" benefits/costs"
26. "Cross-sectoral collaboration and policy making approaches; disaster epidemiology; best practices for local implementation of climate and health related assessments, interventions, and policies; health in all policies; root cause analysis; systems thinking"
27. "Mitigating exposure to environmental toxins."
